# Classification of perovskite structural types with dynamical octahedral tilting

**DOI:** 10.1107/S2052252523002208

**Published:** 2023-03-28

**Authors:** Donat J. Adams, Sergey V. Churakov

**Affiliations:** a University of Bern, Bern, Switzerland; b Laboratory for Waste Management, Paul Scherrer Institute, Villigen-PSI, Switzerland; Alfred University, USA

**Keywords:** perovskites, symmetry mode analysis, structure prediction, hybrid materials, decoupled anharmonic mode approximation, dynamical simulations, octahedral tilting, dynamic disorder

## Abstract

Perovskites *ABX*_3_ with delocalized positions of the *X* atoms represent a distinct class of dynamically distorted structures with peculiar structural relations and physical properties. The delocalization originates from atoms crossing shallow barriers of the potential energy surface. Quantum mechanically, they can be treated similar to light atoms in diffusive states. Many of these perovskite structures are widely used functional materials thanks to their particular physical properties, such as superconductivity, ferroelectricity and photo-activity. A number of these properties are related to static or dynamic motion of octahedral units. Yet, a full understanding of the relationships between perovskite crystal structure, chemical bonding and physical properties is currently missing. Several studies indicate the existence of dynamic disorder generated by anharmonic motion of octahedral units, *e.g.* in halide perovskite structures. To simplify structural analysis of such systems we derive a set of space groups for simple perovskites *ABX*_3_ with dynamical octahedral tilting. The derived space groups extend the well established space group tables for static tiltings by Glazer [*Acta Cryst. B* (1972 ▸). **28**, 3384–3392], Aleksandrov [*Ferroelectrics* (1976 ▸). **24**, 801–805] and Howard & Stokes [*Acta Cryst. B* (1998 ▸). **54**, 782–789]. Ubiquity of dynamical tilting is demonstrated by an analysis of the structural data for perovskites reported in recent scientific publications and the signature of dynamic tilting in the corresponding structures is discussed, which can be summarized as follows: (*a*) volume increase upon a lowering of temperature, (*b*) apparent distortion of octahedra (where Jahn–Teller distortions can be ruled out), (*c*) mismatch between observed instantaneous symmetry and average symmetry, (*d*) deviation of the experimental space group from the theoretically predicted structures for static tilting, (*e*) inconsistency of lattice parameters with those suggested by the theory of static tilts, and (*f*) large displacement parameters for atoms at the *X* and *B* sites. Finally, the possible influence of dynamic disorder on the physical properties of halide perovskites is discussed.

## Abstract 2

Here, a new class of dynamically distorted perovskite structures is proposed. Based on a complete table of all possible structures, experimental data are re-examined and space group relationships are explained. A list of characteristic features of dynamically distorted perovskite structures is also provided.

## 1. Introduction

Interatomic interactions determine the symmetry and stability of crystal structure in minerals and synthetic materials. Specific structural arrangement of the atoms and their interaction are linked to the electron distribution and thermal motion of atoms commonly described by vibrational ellipsoids (Demetriou *et al.*, 2005 ▸). Strong interplay between the motion of atomic cores and electronic structure is particularly relevant when it comes to phonon–electron coupling, which is responsible for many remarkable properties of materials such as ferroelectricity [*e.g.* SrTiO_3_ (Choudhury *et al.*, 2008 ▸)], superconductivity (Bednorz & Müller, 1986 ▸, 1988 ▸) and photoactivity (Iaru *et al.*, 2017 ▸; Zhao *et al.*, 2019 ▸). These electron–phonon interactions are particularly well documented in hybrid halide perovskites (Munson *et al.*, 2019 ▸). Phonon–electron coupling is held responsible for the fact that hybrid halide perovskites materials have particularly interesting applications, including solar cells with particularly high efficiency.

A classical picture of a solid assumes that the atomic nuclei are localized at well defined minima of the potential energy. In many systems, such a minimum can be well described by a harmonic approximation. The quantum mechanical treatment of solids reveals that, even in the limit of *T* → 0 K, the probability density of the nucleons is not point-like but is extended over a finite range (Heisenberg uncertainty principle). Thermal excitations lead to the statistical occupation of higher vibrational energy levels in the corresponding discrete energy spectrum. Eventually the atomic probability density is smeared out over a broad spatial range, conventionally represented as vibrational ellipsoids (see Fig. 2 for how dynamical tilts could explain large vibrational ellipsoids in structures with dynamical tilting).

The amplitude of atomic motion depends on the shape of the potential energy minima and the excitation probability. For light atoms, the spatial extension of the nuclei can be larger, *e.g.* in the case of hopping of hydrogen atoms (Völkl & Alefeld, 1978 ▸). The associated wavefunction is delocalized over a large domain. The potential energy surface (PES) in such a system has several local minima separated by low activation barriers, allowing instantaneous localization of atoms in different positions (Churakov & Wunder, 2004 ▸). In this article, we will consider the systems in which the delocalized state of atoms is responsible for some outstanding macroscopic properties. The physical origin of these phenomena is related to the quantum mechanical description of the atomic motion and requires clear distinction of the PES and total energy of the system, as defined in Appendix *A*1[App appa]. We will also further develop this notion and consider atoms as delocalized.

In crystal structures, the atomic dynamic can be considered as a collective motion of several atoms, rather than dynamics of individual atoms. The resulting collective degrees of freedom are vibro-rotational modes, often addressed as the eigenmodes of the dynamical matrix. We find it particularly appealing to consider rotational modes, which in some perovskite structures are delocalized over a multi-well PES. This can be inherent to some perovskite phases within certain cell geometries.

**Figure 1:**
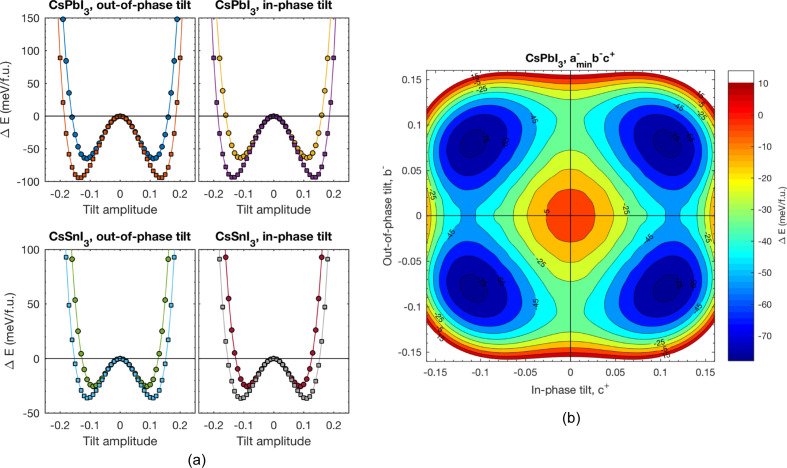
(*a*) Potential energy as a function of tilt amplitude for in-phase and out-of-phase tilts in CsPbI_3_ and CsSnI_3_. Circles and squares in all four panels represent fixed and tetragonally relaxed unit cells, respectively. (*b*) The 2D octahedral tilting PES in CsPbI_3_ with the out-of-phase tilt around the pseudo-cubic axis fixed to its value in the fully relaxed *a* ^−^ *a* ^−^ *c* ^+^ structure. The *x* coordinate axis gives the magnitude of the in-phase tilt around the pseudo-cubic *c* axis, while the *y* coordinate axis gives the magnitude of the out-of-phase tilt around *b* pseudo-cubic axes. From Klarbring (2019 ▸). Reproduced with kind permission from the American Physical Society.

In such structures, atoms are confined by a PES with multiple local energy minima (see *e.g.* Figs. 1 ▸ or 2 ▸) and are separated by small energy barriers [40 meV, for CH_3_NH_3_PbI_3_ (Beecher *et al.*, 2016 ▸), or 20–100 meV in CsPbI_3_ and CsSnI_3_ (Klarbring, 2019 ▸)]. If these barriers are low compared with the thermal energy, the transition between these minima is enhanced and can be excited by small perturbations (Adams & Passerone, 2016 ▸).

Furthermore, we find that dynamic octahedral rotations are ubiquitous in halide perovskites and have actually already been observed, for example in CsPbCl_3_ (Fujii *et al.*, 1974 ▸), CH_3_NH_3_PbBr_3_ and CH_3_NH_3_PbCl_3_ (Chi *et al.*, 2005 ▸; Swainson *et al.*, 2003 ▸, 2015 ▸) – see also Beecher *et al.* (2016 ▸) and Marronnier *et al.* (2017 ▸). This kind of disorder can be characterized as dynamic, *i.e.* atoms instantaneously occupy specific positions but on average these positions are only partially occupied. Related to the observations in the cubic phase of CH_3_NH_3_PbI_3_ through inelastic X-ray scattering, the term ‘dynamic disorder’ has been coined (Poglitsch & Weber, 1987 ▸; Egger *et al.*, 2018 ▸) [see also the review of Whalley *et al.* (2017 ▸)].

Gao *et al.* (2021 ▸) found ‘structural dynamics resembling that of a liquid’, describing a dynamic in a PES with multiple energy minima linked through shallow energy barriers, which can be crossed even at low temperature, either due to thermal excitations or due to quantum tunnelling.

In this article, we will refer to these octahedral rotations as ‘dynamic tilting’. The dynamic instabilities are involved in the series of phase transitions in CH_3_NH_3_PbI_3_: from the orthorhombic phase (*Pbn*2_1_ space group), to tetragonal structure (*I*4*cm* space group) at ∼160 K, and finally to the pseudo-cubic tetragonal phase at ∼330 K (*P*4*mm* space group) (Onoda-Yamamuro *et al.*, 1992 ▸).

**Figure 2:**
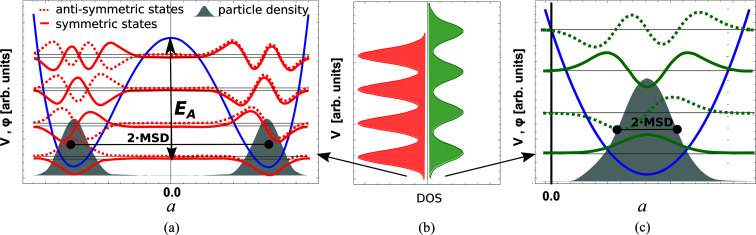
A sketch of the PES *V*(*a*) as a function of the rotational angle *a* of the *BX*_3_ octahedra. The atomic configurations generating these potentials are shown in Fig. 3 ▸. (*a*) Dynamic tilting: the rotational degree of freedom is subject to a double-well potential *V*(*a*). From numerical calculations, the resulting ionic wave function φ(*a*) can be derived. In the case of a double well, states at the lowest energy show two distinct localizations in both potential minima and so does the particle density |φ(*a*)|^2^ (shaded in grey). However, the average tilting angle is at *a* = 0 – *i.e.* in between the two minima. The particle density (grey) is split and thus can explain large atomic MSDs or, according to the representation of the motion, large vibrational ellipsoids in structures with dynamical tilting. The vibrational energy spectrum is not equispaced in this case due to quasi-degeneracy. (*b*) Comparison of the vibrational DOS, double well (red) versus single well (green). For better visibility, smearing was applied. The energy spectrum is particularly dense for the double well, due to almost degeneracy of these energy states. This gives rise to particularly small transition energies and thus potentially to large polarizability. (*c*) Static tilting: the octahedral rotational degree of freedom is subject to a single potential with a minimum at *a* ≠ 0. The resulting ionic wave function φ(*a*) with the lowest energy shows a single localization at the potential energy minimum. The energy spectrum is equispaced.

As previous calculations show (Adams & Oganov, 2006 ▸), such dynamical tiltings can lead to particular phonon-band structures with a high density of states (DOS) at low energies – see Fig. 2 ▸(*a*). These vibrational modes are relevant for the electron–phonon interaction, possibly explaining the unusually high susceptibility of hybrid halide perovskites to external factors such as electric fields, temperature, humidity or mechanical stress (Lin *et al.*, 2021 ▸; Ugur *et al.*, 2020 ▸). The observed properties can be related to the peculiar shape of the PES. Interestingly, it is precisely these instabilities and the resulting dynamic tilting (Zhu & Ertekin, 2019 ▸; Zhang *et al.*, 2015 ▸) that can lead to strong electron-scattering rates, large spectral width, and in turn might be the premise of the high efficiency of perovskite solar cells (Wright *et al.*, 2016 ▸; Herz, 2017 ▸).

We therefore systematically explore symmetry constraints on possible dynamic rotations in the octahedra in perovskite structures. Then we explore the most important experimentally accessible signatures of dynamic tilting. Our hypothesis is that many perovskites exhibit dynamic tilting, especially in the group of halide perovskites. Accordingly, we review the most recent publications reporting dynamic tilting, in order to substantiate our hypothesis. Finally, we discuss the possible implications of dynamic tilting for the physical properties of perovskites such as CH_3_NH_3_PbI_3_, considering the electronic structure, vibrational properties (especially phonon dispersion), the electron–phonon coupling and efficiency in photovoltaics.

## 2. Methods

**Figure 3:**
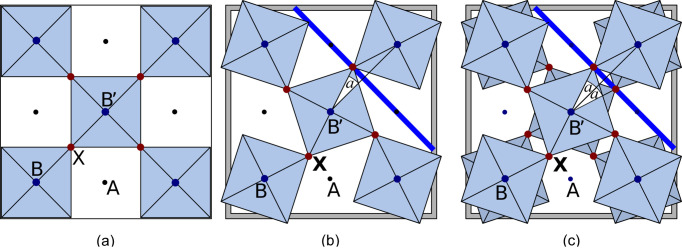
(*a*) Ideal perovskite with untilted octahedra. (*b*) Octahedral tilting by angle *a* allows one to reduce the distance between the cations *B* and *B*′ while the bond length between *B* and *X* is maintained. The result is a contraction of the cell volume (highlighted in grey) due to less volume assigned to the *A* cations. (*c*) Along the tilting axis, two subsequent octahedra can be tilted in antiphase (here, notation *a* ^−^) or in phase [notation *a* ^+^, see (*b*)]. Cross sections of the PES along the blue lines corresponding to octahedral rotations are given in Fig. 2 ▸.

To derive possible tilting systems, a 2 × 2 × 2 supercell of the ideal cubic *AB*O_3_ perovskite containing 40 atoms was set up, in which the rigid octahedra were tilted in three spatial directions. The Glazer notation (Glazer, 1972 ▸) has been used to denote an octahedral tilting by angle *a*, where *a* ^+^ is for two succeeding octahedra tilted in phase and *a* ^−^ is for succeeding octahedra tilted out of phase. These tilts lead to a contraction of the two lattice parameters, which are perpendicular to the tilting axis – see also Fig. 3 ▸. This reduction has been implemented using three-dimensional rotation matrices. They preserve the bonding distance within the octahedron without further (collective) constraints on the structure.

As explained in the *Introduction* [Sec sec1], recent studies have lead to the discovery of dynamic tilts (Marronnier *et al.*, 2017 ▸; Adams & Passerone, 2016 ▸). They result from octahedra confined in the structure by a PES with multiple energy minima – see Fig. 2 ▸. In this study, we only consider systems with symmetric arrangements of minima of the PES relative to a mirror plane. In these structures the tilt angle oscillates between positive and negative amplitude, spending most of the time at |*a*| > 0, whereas the average position is still *a* = 0 – see Fig. 2 ▸. However, due to the non-zero average tilt amplitude, time averages of cell parameters perpendicular to the tilting axis appear shorted compared with the untilted cubic structure. We denote these tiltings by *a* ^*d*^ and consider them by leaving the atomic positions unchanged and contracting the cell parameters perpendicular to the tilting axis.

### 2.1. Classification of dynamic tilt systems

The possible combinations of dynamic tilts and their combinations with static tilts were applied and the resulting structures were classified using the crystallographic tool *FINDSYM* (Stokes & Hatch, 2005 ▸). It determines the primitive unit cell, lattice vectors, the point group of the lattice and thus the unique space group of the crystal structure. For static tilts, Howard & Stokes (1998 ▸) showed that the 25 distinct isotropy subgroups of static tilts can be reduced to 15. They considered only ‘simple’ tilt systems, in which the tilts around a particular axis have the same magnitude and either the same sign (the + pattern) or alternating sign (the − pattern). This results in the exclusion of tilt systems that accidently show the same tilt angle but different types of tilting (+, −) on different axes. From application of this concept to the dynamic tilts, *i.e.* excluding tilting systems showing the same tilting angle but different types of tilting along different axes, 19 different tilting systems have been derived. Their space groups are reported in Table 1 ▸.

## 3. Applications and results

### 3.1. Volume changes across phase transitions and negative thermal expansion

Positive thermal expansion is common for many materials, a property which is maintained even across phase transitions and linked to extended thermal movement upon temperature increase or more precisely to lowering of the free energy upon lattice expansion. A negative thermal expansion is rather unusual and needs clarification.

The explanation of the negative thermal expansion comes naturally when dynamical tilting is considered. Note that any (dynamic or static) tilting leads to a decrease in the unit-cell volume – as long as the octahedra are considered as rigid. This is because within this approximation the volume is proportional to 

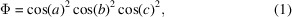

where *a*, *b* and *c* are tilting angles. Thus, Φ has a maximum at *a* = *b* = *c* = 0, and therefore the cubic *Pm* 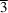 *m* structure usually denoted as the tilting system *a* ^0^ *a* ^0^ *a* ^0^ should have the largest volume, which however is not always observed. In the presence of dynamical tilting, the cubic structure is rather attributed to the *a* ^*d*^ *a* ^*d*^ *a* ^*d*^ tilting system. Therefore, the sharp structural phase transition at 327.15 K in CH_3_NH_3_PbI_3_ from a tetragonal phase to the cubic phase (Jacobsson *et al.*, 2015 ▸), which is linked to a volume drop, can be understood as an activation of additional dynamic tiltings leading to a volume decrease according to equation (1). This explanation is further supported by calculations that show large negative portions of the band structure around the *R* and *M* points of the Brillouin zone corresponding to octahedral tilting (Brivio *et al.*, 2015 ▸; Akbarzadeh *et al.*, 2005 ▸), and observations revealing large thermal movement (Tyson *et al.*, 2017 ▸).

Similar observations are available in other structures, *e.g.* in KNbO_3_. There the volume change is positive across both phase transitions observed between 300 and 750 K (Sakakura *et al.*, 2011 ▸), with the cubic *Pm* 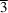 *m* structure formed at high temperature after a series of transitions.

In hybrid halide perovskite semiconductors the presence of organic ions generally reduces the crystal symmetry compared with simple perovskites containing monoatomic ions. Therefore, the space groups listed in Table 1 ▸ cannot be expected to occur there. However, some hybrid halide (Lehmann *et al.*, 2019 ▸; Mante *et al.*, 2018 ▸), such as perovskites, show a (directional) negative thermal expansion or volume change across phase transitions, which can be viewed as a fingerprint of the dynamical tilting.

### 3.2. Agreement between lattice parameters and tilts system

The lattice parameters published *e.g.* by Brivio *et al.* (2015 ▸) for methylammonium lead iodide are in agreement with the underlying tilt system. For the tetragonal *I*4/*mcm* phase, *c*/*a* > 1, while for the orthogonal phase, the calculated *c*/*a* < 1 is consistent with their respective tilt system. In other materials, such as Pr_0.5_Sr_0.5_MnO_3_, which is reported in the *Fmmm* and *I*4/*mcm* symmetry, static tilts cannot explain the pseudo-cubic *c*/*a* ratio of 1.02 (tilt system *a* ^−^ *b* ^0^ *b* ^0^). Static tilt would require rotation of more than 10°, whereas the observed rotation angle is only 3.8°. Consistent results can be obtained by considering dynamic tilts, which leads to modification of the lattice parameters without changing crystal coordinates and resulting in the *Fmmm* space group.

The *Fmmm* structure is also reported for other Pr/Sr ratios (Knížek *et al.*, 2004 ▸) and for combined Pr—Sr—Ce doping (Heyraud *et al.*, 2013 ▸). The Pr sites retain their statistically distributed fractional occupancies and no ionic ordering takes place. It is tempting to explain the discrepancy between tilt system and cell parameters by assuming distortion of octahedral sites. This, however, would undermine the success of the theory of static tilts based on rigid octahedra. Considering dynamical tilting, it is obvious that non-zero average tilt amplitude reduces cell parameters perpendicular to the tilting axis, while the instantaneous geometry of the octahedron remains rigid.

### 3.3. Instantaneous atomic positions and time-averaged structure

Egger *et al.* (2018 ▸) discussed the possibility for dynamic tilting in halide perovskites (‘dynamical disorder’ in their terminology). The arguments for the presence of dynamic disorder were gathered by analysing structural results obtained by complementary experimental methods. While X-ray diffraction yields an averaged structure with high symmetry, Raman spectroscopy shows a local structure with low symmetry. Similarly, Beecher *et al.* (2016 ▸) report ‘anharmonic modes […] with diffusive (order–disorder) dynamics persisting many tens to hundreds of Kelvin above the transition’. Still, as has been stated, these modes are unobservable by Bragg diffraction. Indeed, these simultaneous observations can be reconciled through the concept of dynamical tilting: we interpret these findings as snapshots of the dynamic tilting. It has been stated (Kassan-Ogly & Naish, 1986 ▸) that the multi-well nature of the atomic PES cannot only lead to structural phase transitions to new phases, which is the main subject of this article, but also to diffuse scattering: above the phase-transition temperature the atoms retain some characteristics of the interactions below the phase transition (*e.g.* coupling of the octahedra), which leads to correlated movement of the octahedra.

Gao *et al.* (2021 ▸) experimentally found ‘structural dynamics resembling that of a liquid’ for Cs*M*Br_3_ (*M* = Pb, Sn, Ge) based on diffuse inelastic light scattering that increases towards 0 cm^−1^. This can be generated by atomic dynamics on a PES with multiple energy minima linked through shallow energy barriers. Due to quantum tunnelling or thermal excitations, atoms can cross these barriers, even at low temperature. After tunnelling, there is no classical force to restore the original configuration and the corresponding vibrational excitation frequencies thus tend towards 0 cm^−1^.

The transition rate may vary, depending on the height of the energy barriers between adjacent potential energy minima [this can be described in terms of Fermi’s golden rule for the transition rate: 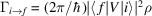 , where *f* and *i* are the final state and the initial state, respectively, ρ is the density of the final states and 〈*f*|*V*|*i*〉 is the matrix element connecting the two states]. A high energy barrier for tilting can result in sluggish dynamics, at a time scale significantly larger than diffraction experiments, making visible the tilting of the octahedra (through the diffuse scattering) in crystals of otherwise higher symmetry. This high symmetry appears in the time-averaged X-ray patterns, or equivalently, in the averaged structure in molecular dynamics.

Similar observations can be made for other structures, such as PbZrO_3_ and Zr-rich PbZr_1−*x*_Ti_*x*_O_3_, which are known to adopt a cubic 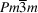 structure above the Curie temperature of *T*_C_ = 523 K (Zhang *et al.*, 2015 ▸) [at room temperature, a centrosymmetric structure (space group *Pbam*) is observed, resulting from antiparallel displacements of the cations on the (110) planes and oxygen octahedral tilts of type *a* ^−^ *a* ^−^ *c* ^0^ (Glazer *et al.*, 1993 ▸)]. At a high temperature of *T* > 523 K, however, the diffraction pattern contains a considerable amount of diffuse scattering, which can be attributed to distortion modes at the *M* point in the Brillouin zone, *i.e.* correlated dynamical in-phase tiltings along the crystal main axis in agreement with molecular dynamic simulations (Zhang *et al.*, 2015 ▸).

### 3.4. Possible space groups for perovskites with dynamical tilting

Often, the structure refinement for perovskites is based on the space groups that are listed in the tables of Glazer (1972 ▸), Aleksandrov (1976 ▸) or Howard & Stokes (1998 ▸), and therefore can be explained by static tilts.

As mentioned already, hybrid halide perovskite structural refinement should not be restricted to these structures, due to the presence of organic ions, which alter crystal symmetry compared with simple perovskites containing monoatomic ions. This has been widely accepted by the research community working with hybrid halide perovskite and helps to avoid wrong crystal symmetry assignment and misinterpretation of phase diagrams.

It is described in the *Methods* [Sec sec2] that dynamical tilting can lead to space groups such as *Fmmm*, *P*4/*mmm*, *Cmmm* and *Pmmm*, which cannot be explained by static tilts. In the following sections (Sections 4.1–4.5), we review published perovskite structures with these space groups and show also that in perovskite CaTiO_3_ and cryolite Na_3_AlF_6_ the known data point towards the presence of dynamical tiltings in these systems.

## 4. Discussion

Dynamic tilting can explain some observed structural phase transitions and important physical properties within the perovskite class of structures. In many compounds, the composition forbids an atomic arrangement corresponding precisely to the perovskite *ABX*_3_ described above. This is often due to the chemical composition involving further chemical species or the ordering of the ions, frequently occurring in a rock-salt-like arrangement of the *A* and *B* cations or of the involved octahedra. Many of the resulting compounds are captured by the structure formula *A*_2_ *B*′*B*′′*X*_6_ and are called double perovskites or layered perovskites. This class retains the stability and often also the rigidity of the octahedra (Hossain *et al.*, 2018 ▸), while more chemical compositions are feasible than in the simple *ABX*_3_ composition. Layered perovskites are of great importance due to their strong and unusual magnetic interactions (Bristowe *et al.*, 2015 ▸), superconductivity (Bednorz & Müller, 1986 ▸), and technical applications (Fan *et al.*, 2015 ▸; Granados del Águila *et al.*, 2020 ▸). We will therefore include some of these double perovskites in the discussion.

### 4.1. *P*4/*mmm*

Many structures are reported in the *P*4/*mmm* crystal structure: BaTiO_3_ (Buttner & Maslen, 1992 ▸) – the name of which is used for the whole crystal class of perovskites with *P*4/*mmm* symmetry – KCuF_3_ and KCrF_3_ (Edwards & Peacock, 1959 ▸), CeAlO_3_ (Tanaka *et al.*, 1993 ▸), TlCuF_3_ (Rüdorff *et al.*, 1963 ▸), SrFeO_3_ (Diodati *et al.*, 2012 ▸), CsAuCl_3_ (Matsushita *et al.*, 2007 ▸), and CeGaO_3_ (Shishido *et al.*, 1997 ▸).

For BaTiO_3_ phase transitions from rhombohedral to orthorhombic, the tetragonal and cubic phases are known (Hayward & Salje, 2002 ▸). All phases except the rhombohedral phase show instabilities along several phonon modes, usually displayed as ‘negative frequencies’, see *e.g.* Fig. 2 of Zhang *et al.* (2016 ▸) or Lebedev (2009 ▸). This indicates that the *P*4/*mmm* structure shows a negative curvature of the PES at zero tilting angle, *e.g.* for the longitudinal optic (LO) 1*E* mode, and the structure therefore might be stabilized by entropy rather than the potential energy.

**Figure 4:**
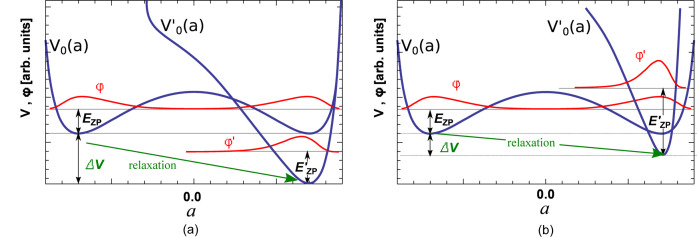
Structure stabilization in the limiting case *T* → 0 K, with classical versus quantum mechanical treatment. The same symbols are used as in Fig. 2 ▸: *a* is the rotational angle of the *BX*_3_ octahedron, *V*_0_(*a*) and *V*_0_′(*a*) are maps of the PES before and after structural relaxation, and red corresponds to ionic wave functions φ(*a*) and φ′(*a*). Structural optimizations of *V*_0_(*a*) → *V*_0_′(*a*) using *e.g. ab initio* calculations (green arrows, ‘relaxation’) lead to a reduction in the minimum of the potential energy Δ*V* (classical treatment). Generally, the symmetry of the structure is reduced by relaxation. (*a*) Quantum mechanical treatment: in the limiting case *T* → 0 K, an additional part of the total energy is due to the kinetic energy – the zero-point energy *E*_ZP_. This scenario could include the transition from *Immm* to *P*2_1_/*n* in cryolite at the critical temperature *T*_C_ = 885 K (Anthony *et al.*, 2005 ▸), but also could include the cubic to *Pmmm* transition in PbTiO_3_-perovskite (Cole & Espenschied, 1937 ▸) between 600 and 800 K (Zhu *et al.*, 2011 ▸), and some of the transitions in CaTiO_3_. Only if *E*_ZP_ before relaxation is comparable to *E*_ZP_′ after relaxation, is it sufficient to compare the minima of *V*_0_(*a*) and *V*_0_′(*a*) to determine the stability. (*b*) If, on the other hand, the reduction of the energy Δ*V* is rather small during the optimization and/or the resulting potential is narrow, the situation arises that Δ*V* < 0 but 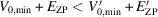 (quantum mechanical treatment), and therefore the high symmetry phase is stabilized even at low temperatures. This kind of stabilization is possible for CeAlO_3_, TlCuF_3_, SrFeO_3_, CsAuCl_3_, CeGaO_3_ CeAlO_3_, TlCuF_3_, SrFeO_3_, CsAuCl_3_ or CeGaO_3_ at low temperatures, as they remain in the *P*4/*mmm* phase.

We would like to stress here that dynamic instabilities do not necessarily indicate a structural instability in the limit *T* → 0 K. In the framework of quantum mechanics, the ground state of the system at *T* → 0 K corresponds to the minimum of the total energy, which is the sum of potential energy *V*_0_ and kinetic energy of the nuclei (referred to as zero-point energy *E*_ZP_) (see Appendix *A*1[App appa] for a detailed definition). Calculations (Adams & Passerone, 2016 ▸) suggest that *E*_ZP_ is particularly small in structures with delocalized atomic configurations represented by a multi-well PES. Therefore, sometimes the total energy (*V*_0_ + *E*_ZP_) can have a minimum at a structure exhibiting a multi-well PES, rather than at a structure corresponding to the minimum of *V*_0_ (see also Fig. 4 ▸ and Appendix *A*2[App appa]).

This important difference between the PES and the total energy could be overseen by atomistic simulations, where often only *V*_0_ is accounted for. Therefore, for the remaining structures reported (CeAlO_3_, TlCuF_3_, SrFeO_3_, CsAuCl_3_ and CeGaO_3_), the stabilization through the zero-point energy is plausible, as they remain stable in the *P*4/*mmm* phase down to low temperatures.

### 4.2. Cmmm

The *Cmmm* space group has been reported for (Li,La)TiO_3_-perovskite systems, which in turn give their name to the crystal class (Sanz *et al.*, 2004 ▸). NaIO_3_ is also known in the *Cmmm* space group (Zachariasen, 1928 ▸), as well as Nd_0.7_TiO_3_ (Sefat *et al.*, 2005 ▸).

### 4.3. Pmmm

Another crystal structure type is *Pmmm* PbTiO_3_-perovskite (Cole & Espenschied, 1937 ▸). At ambient pressure it undergoes a phase transition to the cubic phase between 600 and 800 K (Zhu *et al.*, 2011 ▸). The same space group is also found for NaNbO_3_ (Solovev *et al.*, 1961 ▸), Mg_0.5_W_0.5_O_3_ (Zaslavskii & Bryzhina, 1963 ▸) and GdCoO_3_ (Ruggiero & Ferro, 1954 ▸). NaNbO_3_ particularly attracts our attention. It has been refined recently (Peel *et al.*, 2012 ▸) for the high temperature phase at 773.15 K. Best refinements were achieved with space groups *Pmmm* (χ^2^ = 1.80) and *Pnma* (χ^2^ = 1.85).

### 4.4. Perovskite CaTiO_3_

In CaTiO_3_, a cascade of phase transitions from *Pbnm* to *Cmcm* (1380 K), *I*4/*mcm* (1500 K) and finally to the cubic *Pm* 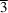 *m* phase (1580 K) is observed. For the first transition (*Pbnm* to *Cmcm* at 1380 K), small anomalies in the temperature dependence of the cell and structural parameters are observed (Kennedy *et al.*, 1999 ▸).

However, other authors mention the large atomic displacement parameter of the cubic phase and state: ‘the high-temperature phase transition to cubic perovskite is triggered by the sudden increase of the mobility of the oxygen sublattice or at least of parts of it.’ (Vogt & Schmahl, 1993 ▸). Quenching allows one to access the dynamic disorder of the cubic phase (Britvin *et al.*, 2022 ▸). Furthermore, within the approximation of static tilts, the fading of tilting angles at the *I*4/*mcm*–*Pm* 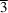 *m* phase boundary at 1500 K – Fig. 4 of Kennedy *et al.* (1999 ▸) – should lead to a significant increase of the volume in a temperature range of 20–50 K, which is not observed. Therefore, the cubic phase should be assigned to a dynamic tilting of oxygen octahedra (*a* ^*d*^ *a* ^*d*^ *a* ^*d*^) rather to an ideal perovskite structure (*a* ^0^ *a* ^0^ *a* ^0^).

### 4.5. Cryolite Na_3_AlF_6_

**Figure 5:**
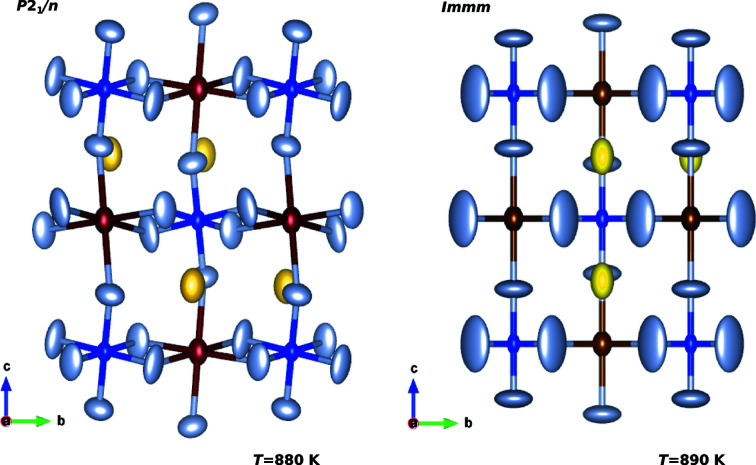
Thermal vibrational ellipsoids in cryolite at *T* = 880 K and *T* = 890 K, *i.e.* below and above the phase transition, respectively, as reported by Yang *et al.* (1993 ▸). Colour code: Al is blue, Na1 and Na2 are red and yellow, and F is grey. At *T* = 890 K in the high temperature phase, neither the atomic positions nor the vibrational octahedra suggest static tilting.

The phase transition is well documented in cryolite (Steward & Rooksby, 1953 ▸; Spearing *et al.*, 1994 ▸). The critical temperature for the transition between the *P*2_1_/*n* space group and the *Immm* space group (Anthony *et al.*, 2005 ▸) is *T*_C_ = 885 K (612°C). Due to excellent X-ray data, the atomic positions and the main axes of the vibrational ellipsoids are known (Yang *et al.*, 1993 ▸) below and above *T*_C_. At low temperature, the system shows static tilts. The *P*2_1_/*n* space group is generated by rigid octahedra and a tilt system with one in-phase tilting and two out-of-phase tiltings – see also Fig. 5 ▸. Due to the rock-salt ordering of the octahedra, the classification for simple perovskites (Lufaso & Woodward, 2001 ▸; Woodward, 1997*a* ▸,*b* ▸) – where *a* ^+^ *b* ^−^ *c* ^−^ corresponds to *P*2_1_/*m* – cannot be applied. The experimental high-temperature orthorhombic *Immm* structure shows displacements of fluorine from the ideal cubic perovskite positions. By further inspection, they result from different bonding lengths in AlO_6_ and NaO_6_ octahedra. The pseudo-cubic lattice parameters, on the other hand, suggest a tilting by at least 2.5°

Finally, the main axes of vibrational ellipsoids can increase by more than 200% over the phase transition. All this points toward a system with at least one dynamic tilting.

### 4.6. Impact of dynamical tilting on the physical properties

The multi-well energy landscape and the amplification of thermal movement upon temperature increase lead to: (*a*) unusually large thermal anisotropic displacement factors for *B* and *X* sites, see *e.g.* Tyson *et al.* (2017 ▸) for CH_3_NH_3_PbI_3_; (*b*) negative (directional) temperature expansion, see *e.g.* Jacobsson *et al.* (2015 ▸) for the negative directional temperature expansion in CH_3_NH_3_PbI_3_; and (*c*) cell-volume decrease at the phase transition upon temperature increase, see *e.g.* Sakakura *et al.* (2011 ▸) discussing the volume contraction in Na_0.5_K_0.5_NbO_3_ at the phase transitions at 446 and 666 K.

Wright *et al.* (2016 ▸) investigated the electron–phonon coupling in hybrid lead halide perovskites in order to access the coupling transport properties, charge-carrier recombination and finally the charge-carrier mobility. It is surprising that the structural instabilities are overseen in these calculations. The harmonic approximation results in imaginary frequencies in these materials [see also Yang *et al.* (2017 ▸)], which is a signature of the negative curvature of the PES. However, the frequencies cannot be interpreted physically. Adams & Passerone (2016 ▸) have shown that dynamic instabilities can lead to high DOS in the vibrational dispersion relation close to ω = 0. Furthermore, the electron–phonon coupling coefficient depends on the atomic mean square deviation (MSD) (Antonius *et al.*, 2015 ▸), which diverges for unstable modes in harmonic theory, and which is overestimated for stable modes in harmonic theory (Adams *et al.*, 2020 ▸). It thus remains to be seen how the re-evaluation of the electron–phonon matrix element will change our picture of the electron–phonon in hybrid lead halide perovskites.

However, the importance of anharmonic vibrational excitations in the stabilization of the different phases of halide perovskites is known. They are accessed through Monte Carlo simulations [see *e.g.* Bechtel *et al.* (2019 ▸) for CsPbBr_3_] or Landau theory, which are computationally costly or semi-empirical, respectively [*e.g.* for CsPbI_3_ (Marronnier *et al.*, 2017 ▸)]. However, the evaluation of the correct vibrational spectrum, *e.g.* in decoupled anharmonic mode approximation (DAMA) (Adams & Oganov, 2006 ▸; Adams *et al.*, 2020 ▸), gives access to the free energy and thus to most physical and thermodynamic properties of the material. In some structures, the formation of dynamic instabilities seems to be fostered by pressure, *e.g.* in CsAuCl_3_ (Matsushita *et al.*, 2007 ▸). In these structures, the evaluation of the free energy could clarify the role of pressure in the structure stabilization.

As mentioned in the *Introduction* [Sec sec1], the multi-well character of the PES results in a high sensitivity of the ionic positions on perturbations. This is also reflected in the electron–phonon coupling constant

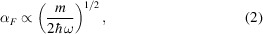

where ℏ is Planck’s reduced constant, *m* is the effective mass of the charge carrier and ω is the frequency of LO phonons (Feynman, 1955 ▸). For degenerate eigenstates, the transition frequency vanishes and the coupling therefore diverges. Hence, the concept of polarons has to be extended.

## 5. Conclusions

### 5.1. Phase stabilization

Dynamic tilts can emerge upon temperature increase. Their mechanism can be explained based on the DAMA model presented by Adams & Passerone (2016 ▸). The double-well PES is the necessary condition for the onset of dynamic tilt (Fig. 2 ▸). The energies of the vibrational modes in the double-well potential are more dense than in a single well [Fig. 2 ▸(*c*), compare also the scale of the energy axis]. Most importantly, the ground state of the double well is almost degenerate, leading to a particularly high DOS at the lowest energy [this high density is calculated by Adams & Passerone (2016 ▸) for the vibrational DOS of cryolite]. Typically, the high temperature phase lies energetically higher than the low temperature phase, *e.g.* Δ*V* = 90 meV per CH_3_NH_3_PbCl_3_ unit has been reported for its cubic phase compared with its orthorhombic phase (Brivio *et al.*, 2015 ▸). This energy difference is outweighed by the relevant thermodynamic potential at temperature, which is the free energy 

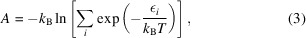

with *k*_B_ as the Boltzmann constant, *T* as the temperature and ε_*i*_ as the energy of the vibrational states. *A* decreases whenever the vibrational energies ε_*i*_ decrease, *e.g.* through lattice expansion, or here when a phase transition leads to a high density of vibrational states at low energies in the spectrum, *e.g.* through degenerate low-energy phonon modes in the cubic phase of CsPbI_3_ (Marronnier *et al.*, 2017 ▸).

The activation energy, *i.e.* the energy required to surmount the energy barrier between different energy minima, is generally comparable with the energy of thermal vibrations at the transition temperature [see Fig. 2 ▸(*a*)]. It is particularly low for structures with small tilting, which, according to Glazer (1972 ▸), appears mostly in structures where the size of the *A* cation matches that of the cavity. This leads to a small tilting and thus a low energy barrier with relatively low transition temperatures [*e.g.* 7.3 meV in CsPbI_3_ in the cubic phase, which transforms to the tetragonal β-phase at 533.15 K (Marronnier *et al.*, 2017 ▸)]. If the *A* cation is small (*e.g.* MgSiO_3_), the tilting is larger, and the phase transition can potentially occur at higher temperatures. This type of transition is expected to be very common. In some systems, however, the critical temperature lies above the melting point and therefore such transition is not observed.

### 5.2. Group–subgroup relations

In general, the appearance or disappearance of a dynamic tilting mode does not lead to the simple group/subgroup relation between resulting structures. This can be shown in the example of the 

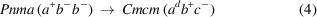

transition, where one *b* ^−^ transforms to the *a* ^*d*^ dynamic tilt. Considering group–subgroup relations, at least one intermediate subgroup is involved (either *Pbcm* or *Pmmm*) [data from Bilbao Crystallographic Server (Aroyo *et al.*, 2006 ▸)]. In other structures, the onset of dynamic tilt does not lead to space group change, *e.g.* in the case of 

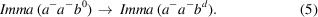

The shortening of the crystal axis related to the onset of the dynamic tilt does not break any symmetry. As shown in these two examples, dynamic tilts do not correspond to irreducible representations of the space group, *i.e.* the two end members relate in a simple or more complex way.

### 5.3. Crystallographic consequences of dynamical tilting

As shown in the few examples, the particular relationships in the structures at phase transition can be indicative for transformations driven by dynamic tilts. Dynamic tilt should be considered if:

(i) the observed instantaneous symmetry is in contradiction with the average symmetry;

(ii) the experimentally observed space group of a perovskite-type structure is not listed in the tables established by Glazer (1972 ▸), Woodward (1997*a* ▸) or Aleksandrov (1976 ▸);

(iii) the proportions of the lattice parameters are in contradiction with those suggested by the theory of static tilts, *e.g.* in the *I*4/*mcm* symmetry, which appears in the *a* ^*d*^ *a* ^*d*^ *b* ^−^ system as well as the *a* ^0^ *a* ^0^ *b* ^−^ tilting system, the first one corresponds strictly to a lattice ratio of *c*/*a* > 1 while the second one has no restrictions for this ratio;

(iv) the octahedra appear distorted. The octahedra represent a stable configuration of the *BX*_6_ chemical configuration. (Large) distortions are unlikely, except in the case of Jahn–Teller distortions.

The success of the theory of static tilts in numerous other systems (Aleksandrov, 1976 ▸; Aleksandrov & Bartolome, 1994 ▸; Glazer, 1972 ▸; Woodward, 1997*a* ▸,*b* ▸) underlines the correctness of these considerations.

### 5.4. Dynamical tilting and electron–phonon interaction

The interaction between phonons and charge carriers is important for the physical mechanisms controlling the mobility of the charge carriers. High mobility is desirable for applications that are based on the efficient separation of electrons and holes, *e.g.* in photovoltaics. It is significantly influenced by the interaction between photons and the charge carriers. Steele *et al.* (2019 ▸) confirm a strong electron–phonon coupling in CH_3_NH_3_PbCl_3_ and relate it to ‘strong anharmonicity and dynamic disorder’. Similar results are found for CsPbBr_3_ and CH_3_NH_3_PbBr_3_ (Sendner *et al.*, 2016 ▸). In the work of Batignani *et al.* (2018 ▸), the interdependence between dynamic structural distortions, photo-carriers and photons in lead halide perovskites is also documented. The effect of this coupling on the mobility needs further investigation because at large coupling its temperature dependence can exhibit multiple extrema (Prodanović & Vukmirović, 2019 ▸). Such a coupling can have further unexpected effects on the photoluminescence, such as the up-conversion, *i.e.* an increase of the luminescence frequency through energy transfer from phonons (Granados del Águila *et al.*, 2020 ▸).

Based on available observations, we can conclude that dynamical tilting can affect the following properties in perovskites, especially in lead halide perovskites:

(1) The electron–phonon coupling. The electron–phonon coupling coefficient depends on the atomic MSD, which is significantly increased in structures showing dynamical tilting compared with structures without structural instabilities.

(2) The charge-carrier mobility. Amongst others it is limited by the interaction of charge carriers with crystal vibrations (Herz, 2017 ▸). This interaction can be described in terms of polarons, which consists of the polarization of the ionic lattice by a mobile electron (Fröhlich, 1954 ▸; Feynman, 1955 ▸). The resulting field finally creates *e.g.* the Fröhlich interaction. As mentioned above, in a structure with dynamic tiltings the ionic eigenstates of a double well of the PES are degenerate, which results in high sensitivity of the ionic positions on perturbations, and presumably large electron–phonon coupling constant and polarizability.

(3) The spectral width of light-emitting semiconductor devices (Iaru *et al.*, 2017 ▸) by electron–phonon interactions. Wright *et al.* (2016 ▸) underline that the ‘Fröhlich coupling to LO phonons is the predominant charge-carrier scattering mechanism in hybrid lead halide perovskites’, leading to emission linewidth broadening – see also Herz (2017 ▸).

All these properties seem to be tightly related to the observed instability on the surface of the potential energy and the resulting dynamical tilting. Therefore, the inherent structural sensibility might be the premise for the high efficiency of perovskite solar cells, which indicates that the challenge in the application of these materials is the stabilization of the structure against phase transitions. Temperature stabilization of CSPbI_3_ (Kirschner *et al.*, 2019 ▸) points to stabilization through the free energy [see also equation (3)], and thus supports our model.

Many perovskite structures show interesting properties such as photoelectricity, but also magnetism, ferroelectricity or superconductivity. The dynamical tiltings result in almost degenerate vibrational modes (Adams & Passerone, 2016 ▸), which are worth further investigation. These modes could couple to the electronic states and thus modify the electron–electron interaction in the solid in an unexpected manner. For photoelectric materials, Marronnier *et al.* (2017 ▸) explicitly state that ‘the perovskite oscillations through the corresponding energy barrier could explain the underlying ferroelectricity and the dynamical Rashba effect predicted in halide perovskites for photovoltaics’. A number of physical properties of perovskite structures are yet to be explained. The space groups for dynamic tilting of cubic perovskites reported in this work will facilitate consideration of dynamical tilting in connection with physical properties of perovskites. 

## Figures and Tables

**Table 1:** 19 perovskite-type structure tilt systems with dynamic tilting The notation of Glazer (Glazer, 1972 ▸) was extended by a dynamic tilt *a* ^*d*^, indicating that the corresponding octahedra oscillate between two positions (positive and negative amplitude). This tilt can be observed instantaneously and locally as the octahedra appear untilted when averaged over time or/and space. The unit cell is given in terms of the pseudo-cubic axes *a*_p_, *b*_p_ and *c*_p_, which are parallel to the axes of the tilting by angles *a*, *b* and *c*, respectively. The distortion of the pseudo-cubic axes is given by 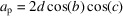 , 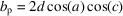 and 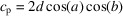 , where *d* corresponds to the *B*—*X* bond distance.

Tilt system	Space group	No.	**a**	**b**	**c**
*a* ^−^ *b* ^−^ *c* ^*d*^	*C*2/*m*	12	2*a*_p_	−2*c*_p_	−*a*_p_ + *b*_p_
*a* ^+^ *b* ^*d*^ *c* ^+^	*Immm*	71	2*a*_p_	2*b*_p_	2*c*_p_
*a* ^*d*^ *b* ^+^ *c* ^−^	*Cmcm*	63	−2*a*_p_	2*c*_p_	2*b*_p_
*a* ^−^ *b* ^*d*^ *c* ^*d*^	*Fmmm*	69	2*a*_p_	2*b*_p_	2*c*_p_
*a* ^*d*^ *b* ^+^ *c* ^*d*^	*Cmcm*	65	−2*a*_p_	2*c*_p_	*b*_p_
*a* ^*d*^ *b* ^−^ *c* ^*d*^	*Fmmm*	69	2*a*_p_	2*b*_p_	2*c*_p_
*a* ^+^ *b* ^*d*^ *c* ^*d*^	*Cmmm*	65	2*c*_p_	−2*b*_p_	*a*_p_
*a* ^*d*^ *b* ^*d*^ *c* ^*d*^	*Pmmm*	47	*c*_p_	*b*_p_	−*a*_p_
*a* ^−^ *a* ^−^ *b* ^*d*^	*Imma*	74	*a*_p_ − *b*_p_	−2*c*_p_	*a*_p_ + *b*_p_
*a* ^+^ *a* ^+^ *b* ^*d*^	*I*4/*mmm*	139	2*a*_p_	2*b*_p_	2*c*_p_
*a* ^*d*^ *a* ^*d*^ *b* ^−^	*I*4/*mcm*	140	−*a*_p_ − *b*_p_	*a*_p_ − *b*_p_	2*c*_p_
*a* ^*d*^ *a* ^*d*^ *b* ^+^	*P*4/*mbm*	127	*a*_p_ + *b*_p_	−*a*_p_ + *b*_p_	*c*_p_
*a* ^*d*^ *a* ^*d*^ *b* ^*d*^	*P*4/*mmm*	123	*b*_p_	*a*_p_	−*c*_p_
*a* ^*d*^ *a* ^*d*^ *a* ^*d*^	*P* 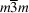	221	−*b*_p_	*a*_p_	*c*_p_
*a* ^0^ *a* ^−^ *b* ^*d*^	*Fmmm*	69	2*a*_p_	2*b*_p_	2*c*_p_
*a* ^0^ *a* ^+^ *b* ^*d*^	*Cmmm*	65	−2*a*_p_	2*c*_p_	*b*_p_
*a* ^0^ *a* ^*d*^ *b* ^*d*^	*Pmmm*	47	*c*_p_	*b*_p_	−*a*_p_
*a* ^0^ *a* ^*d*^ *a* ^*d*^	*P*4/*mmm*	123	*c*_p_	*b*_p_	−*a*_p_
*a* ^*d*^ *a* ^0^ *a* ^0^	*P*4/*mmm*	123	*c*_p_	*b*_p_	−*a*_p_

## Appendix A Structure stability

### A1. The potential energy surface

In this article, we define the potential energy of an atomic configuration (*V*_0_) as the sum of the kinetic energy of the electrons (*T*_e_), the potential energy due to the interaction of electrons (*U*_ee_), the Coulombic energy of electron–nucleus attraction (*V*_ne_) and the energy of interactions between nuclei (*V*_nn_), *i.e.*

For a given atomic configuration, this quantity is readily obtained by widely used *ab initio* codes such as *VASP* (Kresse & Furthmüller, 1996 ▸) or *Quantum ESPRESSO* (Giannozzi *et al.*, 2009 ▸).

Often, the cohesive energy is estimated from the difference of *V*_0_ between different atomic configurations. These estimations correspond to *T* = 0 K and they most often neglect the kinetic energy of the nuclei, which at *T* = 0 K is called the zero-point energy *E*_ZP_.

Minimization of *V*_0_ with respect to atomic arrangement and lattice parameters in the structure is used for prediction of structure stability in the limit of *T* → 0 K, assuming that the atoms behave as classical particles. However, in the framework of quantum mechanics, the stable atomic arrangement at *T* = 0 K corresponds to the minimum of the total energy, which is defined as the sum of *V*_0_ and the kinetic energy of the nuclei, referred to as the zero-point energy *E*_ZP_ (see Appendix *A*2[App appa]).

### A2. Quasi-harmonic approximation

The quasi-harmonic approximation is a widely used reference model for the description of thermodynamic and elastic properties of solids (Palumbo & Dal Corso, 2017 ▸; Togo & Tanaka, 2015 ▸). In this model, low-energy vibrational excitations are described through polarization vectors, which account for the contribution of individual atoms to a collective movement at a fixed lattice constant. In the harmonic approximation, the polarization vectors are chosen in such a way that the second-order interactions between modes vanish. Specifically, in perovskite structures, some polarization vectors correspond to rigid-body-like rotation of octahedra. The vibrational spectra of the system depend on the curvature of the PES along these polarization vectors.

The vibration spectra

where *i* is an integer and

are determined along the degrees of freedom (Griffiths, 1995 ▸) – see also Fig. 2 ▸(*c*). If  , the frequency ω becomes imaginary and the harmonic approximation breaks down.

At *T* → 0 K, the stability of the system consisting of nuclei and electrons is defined by the minimum of the total energy of the system, which is different from the minimum of the potential energy *V*_0_. The difference between these entities is the zero-point energy, *E*_ZP_ = ℏω/2 with ω from equation (8). The value of *E*_ZP_ is thus determined by the shape of the PES in close vicinity to the equilibrium arrangement of atoms in the structure. In the harmonic approximation, *E*_ZP_ is related to the curvature of the PES. Independently of the approximation, the following rule holds: the wider the potential, the smaller the zero-point energy and the closer the total energy to the minimum of the potential energy. *E*_ZP_ is particularly small in the multi-well PES. This can lead to the stabilization of delocalized configurations with partitioning of the atomic density between multiple minima of the PES (see also Fig. 4 ▸), even if the minimum of the potential energy *V*_0_ of the single-well potential is lower than the minimum of the multi-well potential.

### A3. Statistical physics

The quasi-harmonic approximation is widely used to calculate the free energy of solids based on the energy spectrum of the phonons. For the sake of simplicity, the phonon dispersion (*k*-point sampling) can be accounted for by considering sufficiently large supercells. The energies of the spectra [equation (7)] can be used to obtain the partition function

which in turn allows us to calculate the free energy in equation (3). Using the spectrum [equation (7)] and the partition function *Z*, it is possible to calculate most of the thermal properties of a material. As temperature increases, the higher vibrational energy levels influence the material properties. The free energy is the thermodynamic potential, which is minimized by the atomic configuration at *T* > 0 K. Structures showing spectra with low phonon frequency energies minimize ε_*i*_ and thus also *A*. One implication of this dependence is thermal expansion, where larger lattice parameters lead to weaker interactions of atoms and thus also affect the energy spectrum of atomic vibrations (red shift). Therefore, the increasing cell parameters minimize *A* when *T* increases (Guyot *et al.*, 1996 ▸). On the other hand, it has been shown using the example of cryolite that anharmonic effects can lead to a quasi-degeneracy of numerous low-energy states, resulting in a decrease of free energy and structural stabilization. It is clear that this effect plays an important role in stabilization in various perovskites that show a negative curvature of the potential surface near the equilibrium structure.
